# Legal Instructional Design by Deep Learning Theory Under the Background of Educational Psychology

**DOI:** 10.3389/fpsyg.2022.917174

**Published:** 2022-07-19

**Authors:** Zhitao Shen, Shouzheng Zhao

**Affiliations:** School of Law, Shanghai International Studies University, Shanghai, China

**Keywords:** educational psychology, deep learning, instructional design, law teaching, comprehensive quality

## Abstract

This work aims to reform legal teaching in Colleges and Universities (CAUs) and improve law students’ comprehensive quality. In the context of Educational Psychology (EPSY) research, Deep Learning (DL) theory is integrated into legal instructional design (ID). Following a theoretical review of EPSY and DL, the current situation and problems of college legal teaching are understood based on the Law School in a University in Shanghai through auditing, communication, and investigation methods. The theoretical research results are integrated into the ID. The teaching content is divided into language information module, wisdom skills module, cognitive module, action skills module, and attitude module. Each module is divided into three teaching methods, and all teaching methods are combined into the proposed legal ID. Finally, the proposed legal ID is applied in the legal classroom of the Law School in a University in Shanghai. Overall, seventy students are recruited and grouped into Class A (experimental group) and Class B (control group). Class A uses the proposed legal ID, and Class B does not. The scores of Classes A and B are compared. After a semester, the average score of Class A has increased from 68 to 71.11 points. The covariance has decreased from 61.66 to 51.42. When the confidence level is set to 0.95, the confidence interval of class A has increased from 65.26–70.74 to 68.62–73.61. By comparison, the average score of Class B dropped from 68.14 to 68.11 points. The covariance has decreased from 60.24 to 41.76. When the confidence level is set to 0.95, the confidence interval of class B has changed from 65.44–70.85 to 65.86–70.37, without significant improvement. Therefore, the proposed legal ID based on DL theory is scientific and effective. This work has certain reference significance for optimizing the ID of CAUs and improving the comprehensive quality of college-student talents.

## Introduction

With the implementation of the national Rule of Law (RoL) policy, the socialist legal system matures. The law has penetrated all aspects of work, study, and life. Nowadays, legal work is facing many new situations, new problems, and new tests. Today’s society needs high-quality legal talents with comprehensive quality and innovative spirit. College students have gradually become the main force in China’s modern socialist construction. As an essential part of higher education, Colleges and Universities (CAUs) shoulder the important task of cultivating applied and technical talents, which serve in the first line in China (Yu et al., 2021). As the successors of the construction and development of the socialist cause, college students in China should learn professional and practical skills. Also, they must constantly enhance their RoL concept, legal quality, and professional legal knowledge. Such comprehensive learning is vital in constructing socialist RoL and socialist modernization in China. Meanwhile, it is conducive to the harmonious campus construction and the improvement of the comprehensive quality of Chinese college students and provides a strong guarantee for their career development (Ma, 2020).

For instructional design (ID), Liu (2021) systematically analyzes the process of ID, which is mainly divided into six steps: stating objectives, analyzing tasks, determining students’ original level, designing curriculum teaching activities, teaching, and evaluation (Liu, 2021). Mao and Zhang (2020) developed the four basic problems of ID from analysis, selection, and decision-making to the development and evaluation of ID. They combined them with the management process and design motivation to complete the whole process of ID (Mao and Zhang, 2020). Although there are many studies on ID in China, there is little research on Legal ID. From the perspective of constructing the talent training mode of law education, the talent training objectives of law education need to be directionally adjusted (Marzano et al., 2021). Therefore, from Educational Psychology (EPSY) perspective, this work studies the ID of law through the Deep Learning (DL) theory.

**FIGURE 1:**
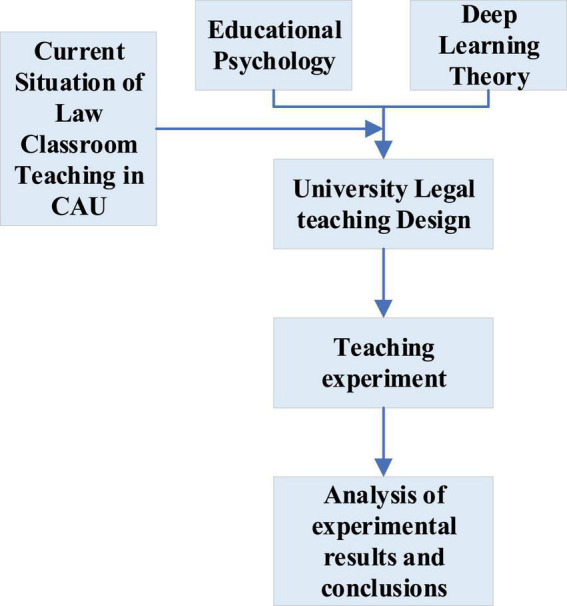
Organization structure of this work.

The purpose is to improve the comprehensive quality of college students and cultivate high-quality legal talents in China. The organizational structure of this work is shown in Figure 1.

Figure 1 shows the organizational structure of this work. It first studies the theory of EPSY and DL. Then, students from the Law School in a University in Shanghai are taken as the research objects to investigate and analyze the current teaching status of law classrooms in CAUs. Then, the problems and underlying reasons are deeply analyzed. As a result, a new legal ID is proposed and applied in the college legal classroom. Finally, comparing the experimental and control groups’ results proves that the proposed legal ID based on the deep learning theory is scientific and effective. These conclusions have certain reference significance for optimizing legal teaching in CAUs and improving the comprehensive quality of law students.

## Current Situation and ID of Law Classrooms in Colleges and Universities

### Educational Psychology

EPSY is the study of human learning in educational contexts, the effects of educational interventions, the psychology of teaching, and the social psychology of school organization. The focus of EPSY is to apply psychology theories to education. These theories can be applied to design curricula, improve teaching methods, promote learning motivation, and help students face various difficulties and challenges (Qian et al., 2018). EPSY is the theory and strategy of studying the effects of memory, cognitive processes, and individual differences on the human learning process. The main task is to guide the basic psychological laws of students’ learning and teachers’ teaching. It reveals learning laws and uses them to promote student learning (Chen, 2019) effectively. EPSY is developed around the interaction process of learning and teaching. The learning-teaching interaction is a systematic process with five elements: students, teachers, teaching content, teaching media, and the teaching environment. It is intertwined by three active processes: the learning process, the teaching process, and the evaluation-reflection process (Liu S. et al., 2021). Students’ learning is no doubt affected by their internal psychological factors, such as learning ability, motivation, and strategies, and external factors such as families, schools, textbooks, and teachers’ teaching. EPSY studies the psychological mechanisms and laws that how these factors affect learning (Xu et al., 2021).

The research field of EPSY mainly includes four aspects: learning psychology, teaching psychology, teacher psychology, and school management psychology. Among them, learning psychology is the most studied and fruitful field, including the following six aspects. 1. Knowledge learning: knowledge is an integral part of students’ learning, and knowledge acquisition is one of the key contents in learning psychology. The connotation of knowledge, the types of knowledge, the characteristics of knowledge, different types of knowledge and learning processes, as well as the conditions restricting learning are specifically elaborated from the perspective of information processing in modern cognitive psychology. 2. Skill learning: skills are also important in students’ learning. Skills are mainly divided into action skills and intelligence skills. Therefore, the research on the formation of skills includes understanding the theory of action skills and formation and the training ways and methods of the two skills. 3. Moral learning: students not only need to acquire knowledge from books to develop skills. At the same time, they must form good moral character. Moral psychology is a traditional research field of EPSY. This field includes moral character and its psychological structure, the analysis of the formation process of moral character, development factors, and training ways and methods. 4. Problem-solving and creativity cultivation: problem-solving ability and creativity are important qualities that students should have. This part of the research involves the psychological description of problems and the process of problem-solving, the factors affecting problem-solving and the cultivation of problem-solving ability, the concept of creativity, and the ways and methods of cultivating creativity. 5. Learning transfer: this field includes the concept and types of learning transfer, the measurement of learning transfer, various traditional and modern theories about learning transfer, the influencing factors of learning transfer, and teaching principles. 6. Learning strategies and motivation. This is one of the most important factors affecting students’ learning. Learning motivation research is an essential link to psychological research. It includes the concept and types of learning motivation, the impact of learning motivation on learning, various theories of learning motivation, and practical ways and specific methods to cultivate and stimulate students’ learning motivation (Zkubat and Zmen, 2021).

Educational Psychology is an integral part of educational theory and technology, which helps to improve teachers’ academic literacy. Meanwhile, it can strengthen teachers’ ability to solve practical problems in education. It allows teachers to understand students more deeply and improve the pertinence of education and teaching. Therefore, by learning EPSY, teachers can deeply understand the psychological basis of relevant teaching measures, enrich teaching art, and comprehensively improve the quality of teaching (Zheng et al., 2018). Moreover, the EPSY can improve dialectical materialism and the consciousness of teachers’ self-education. It is conducive to better Ideological and Political Education (IAPE). It is helpful for teachers to summarize work experience and consciously carry out educational and scientific research (Tai and Chen, 2021).

### Deep Learning Theory

Under the current educational development trend, DL thinking mode has become an irresistible trend. DL, in education, advocates active, critical, and meaningful learning, and its generation and development have a long ideological origin and rich theoretical foundation (Lima et al., 2020). Individuals can only generate DL thinking mode through their experiences and understanding of the world and real life. With DL thinking, they can actively create and construct new knowledge in practice and integrate it into the original cognitive framework to form a new one (Liu Y. et al., 2021).

Constructivism provides an epistemological basis for the development of DL. The core view of Constructivism emphasizes that knowledge is actively constructed by cognitive subjects rather than passively absorbed. Learning will become a meaningful construction process if students can associate the new knowledge with the original one. Under the guidance of this theory, DL pays more attention to learners’ prior knowledge and emphasizes learners’ adaptive expertise (Lauermann and Berger, 2021). Situational cognition provides deep learners with a context for applying knowledge. The core view of Situational Cognition Theory is that knowledge can be gradually understood and further developed only in real life and production activities. Situational Cognition Theory emphasizes that learning can be given real meaning only when learning is embedded in the relevant social or natural context. Then, meaningful learning can enable learners to understand the conditions for applying knowledge and quickly find solutions to problems (Zhang and Zhang, 2020). Distributed cognition provides a methodological basis for the generation and deepening of DL for learners. DL thinking mode emphasizes the influence of context on learners. Deep learners visualize knowledge during communication and convey it to others. Cognitive tools can help learners think deeply and promote higher-order thinking (Apeh et al., 2020). Metacognition monitors and reflects on learners’ DL processes. It urges learners to monitor their cognitive processes and adjust strategies in time to understand and acquire knowledge in learning and life. It also encourages learners to evaluate and reflect on their cognitive outcomes and performance abilities. This promotes overall understanding and increases the degree to which students transfer knowledge to new situations (Nichols, 2020).

### Current Situation of Law Classroom Teaching in Colleges and Universities

The Law School in a University in Shanghai is selected as the research object. Through three-month lectures, classroom observation, and after-class communication with teachers and students, there are some problems in the current situation of law classroom teaching:

(1) Students can be roughly divided into four categories: shallow learning, selective learning, hard-working, and DL. Shallow learning students are interested in law, have a high learning enthusiasm, and listen carefully in class but are reluctant to do homework and lack deep thinking. Thus, they often perform poorly in terms of academic scores. Selective-learning students only learn their points of interest and are disinterested in conceptual and logical reasoning. Hence, they show less concentration. By comparison, hard-working students do their best both in and after class. They actively ask for teachers’ help and try to solve problems through what they learned. Nevertheless, they lack scientific strategies and logical thinking abilities. Thus, their academic performance is moderate. Lastly, a few top students have mastered the DL thinking mode. They concentrate in class, complete homework independently, and achieve good test scores.
(2) Not all teachers correctly understand the teaching reform. Thus, the teaching reform is mostly superficially, without substantial breakthroughs. Some teachers have tried to make reforms, carefully prepared IDs, and grasped students’ interests. However, influenced by traditional concepts, they still ignore students’ subject status and value. In teaching, they focus on transmitting knowledge and have not improved students’ thinking, learning methods, and abilities. Such reforms have made some progress but are still incomplete. Only a small number of teachers have grasped the core of the reform, carried out the reform seriously, and achieved some results. Educational reform cannot be completed overnight but requires perseverance and the support of many educational resources. Therefore, these teachers have also been under tremendous pressure and challenge to persist and promote the teaching reform.
(3) CAUs lack relevant measures and support for practical teaching. Practical teaching is essential for abstract law courses, which can strengthen students’ understanding, memory, and application. Although some social practice courses have been arranged, the specific content is simple and scattered, hardly improving students’ practical legal knowledge. Meanwhile, most social practices are not based on a standardized platform, so the theoretical courses cannot be fully extended. Additionally, relevant rules and regulations for students to participate in practical teaching are not perfect. Most students’ practical courses are only used to collect credits and cannot play a practical role.

### University Legal Instructional Design

**FIGURE 2:**
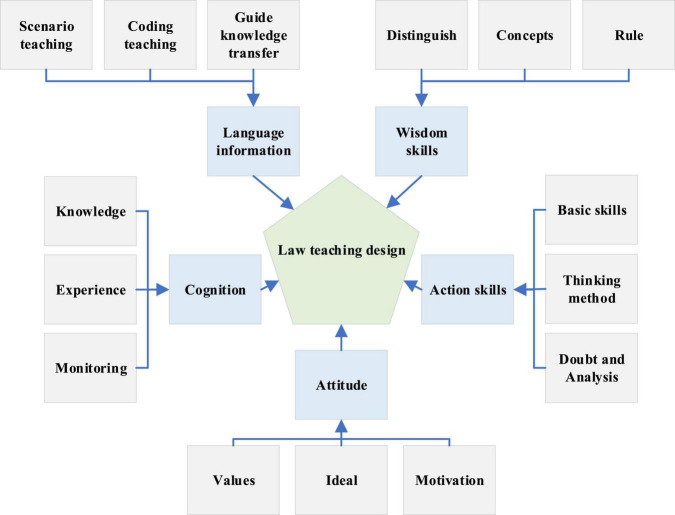
Detail of legal instructional design.

A new DL-based legal ID is formulated according to the problems and reasons found in the current situation of college legal teaching. The ID is divided into five modules: language information module, wisdom skills module, cognitive module, motor skills module, and attitude module (Mehnehj et al., 2020; Njoka and Julius, 2021), as shown in Figure 2.

In Figure 2, the main ways of implementing the language information module include situational teaching, effective coding of legal language information, and guiding students to transfer knowledge. The reasons and purpose of each ID are detailed in Table 1.

**TABLE 1:** Contents of the language information module.

Name	Method	Reason
Language information module	Situational teaching	The conceptual characteristics of law will lead to the abstract content of some textbooks and a lack of intuitive expression. Thus, students cannot easily understand. Besides, the content involves civil, criminal, economic, political, and other aspects, so the legal knowledge is obscure and complex due to the “comprehensive” characteristics of the law.
	Efficient encoding of legal language information	The learning process begins when students pay attention to external situations. Therefore, intuitively encoding newly acquired stimuli is crucial. Learners should be equipped with some information processing methods to make the learned information easy to remember and transfer it to various situations that learners will encounter in the future (Klepsch and Seufert, 2020).
	Guide students to transfer knowledge	To help students master legal concepts, the teacher should teach stress the knowledge points multiple times in class, and additional review and practice are needed. The interaction between old and new legal points is the transfer of legal knowledge (Wati and Widiansyah, 2020).

The wisdom skills module requires teachers to combine legal knowledge with basic skills and cultivate students’ ability to actively acquire knowledge and transform knowledge into skills (Zhang et al., 2021). The specific content is listed in Table 2.

**TABLE 2:** Contents of wisdom skills module.

Name	Method	Meaning
Wisdom skills module	Discriminating learning	Discrimination learning refers to memorizing the characteristics of a legal concept and identifying this concept from a set. It is the simplest kind of learning. The essence of discriminative learning is intuitive learning that differentiates knowledge (Zhu et al., 2021).
	Concept learning	Conceptual learning is the ability to categorize things. Concepts can be abstract or concrete. In particular, concrete concepts are generalized from observations. Abstract concepts are generally learned through definition.
	Rule learning	The essence of rule learning is to apply rules in dynamic situations to transform the rules from the stated form into the procedures that govern people’s behavior.

The purpose of the cognitive module is to allow students to regulate their learning, memory, and thinking to promote their consciousness, initiative, and subjectivity (Vatandoost et al., 2020). The main contents of the cognitive module are stated in Table 3.

**TABLE 3:** Contents of cognitive modules.

Name	Way	Meaning	Contents
Cognitive module	Legal metacognitive knowledge	Students’ cognition of their own legal cognitive process and results; cognition of their own individuals; cognition of legal course learning; cognition of legal learning methods	It means a correct understanding of one’s abilities, strengths and weaknesses, personality, and hobbies. It also includes knowledge of legal study tasks, textbooks, legal knowledge, logical thinking, and learning objectives; knowledge of the learning methods can be selected according to the tasks and objectives.
	Legal metacognitive experience	The emotional experience of students in the process of legal cognitive activities	If students have mastered all the knowledge points of a particular lesson, they will feel happy, excited, and relaxed.
	Legal metacognitive monitoring	Students monitor and adjust their problems in legal cognitive activities	Before students learn a new lesson and make new cognitions, they will develop a complete learning plan for the learning objectives of this lesson. They then continuously adjust their strategies as they learn. Finally, students compare learning goals and learning outcomes to do remedial work.

The action skills module helps teachers teach students to master multiple thinking and learning skills to improve and expand their skills continuously. It guides students to summarize the case information from different perspectives and see the essence through appearance (Guzmán and Joseph, 2020). The specific content is signified in Table 4.

**TABLE 4:** Contents of action skills module.

Name	Target setting	Contents
Action skills module	Basic skills	It refers to the skills of learning legal knowledge, such as interpreting case information, obtaining case materials, judging the value and authenticity of materials, the accuracy of elaborating and explaining legal terms, and the logic of the expression.
	Way of thinking	It includes the cognitive methods of comparison, synthesis and evaluation, and the basic way of thinking combining concepts and cases.
	Doubt and analysis	It includes thinking independently and discovering problems, as well as the rationality of questioning and the feasibility of explaining ideas, and the ability to analyze and think comprehensively.

Attitude is learning to acquire relatively stable internal tendencies and states. Such tendencies and states influence the actions that individuals take about things, people, and times (Orlowski and Girard, 2019). The specific content of the attitude module is demonstrated in Table 5.

**TABLE 5:** Contents of attitude module.

Strategy	Method	Requirements
Attitude strategy	Value judgment	Value judgments are based on the behaviors and thoughts of the characters in the case from the objective reality perspective. Students can grasp the judgment standards of the law and establish relatively correct values.
	Study ideal	Teachers should infiltrate the value and significance of law into students in teaching so that students can deeply understand that through learning law. They can improve their legal awareness, cultural quality, and moral cultivation and promote their all-round development. Additionally, students can fully realize that ideals are the driving force that motivates individuals to study actively and independently and establish a correct learning attitude. Teachers should guide students to consciously link their growth and learning with the country, society, and collective progress.
	Motivation to learn	Teachers should correct students’ wrong and low law study motives and stimulate and cultivate correct study motives. They also guide students to learn how to regulate their law study motives and actively adjust their self-learning motives quickly.

The ID proposed is formulated after consultation and discussion with school leaders and education experts according to students’ problems. Before practical application, it is necessary to change the concept of teachers and students and put forward the student-centered teaching concept. Under this concept, teachers change from authoritarian managers to guides for students, and students change from passive acceptance to active inquiry (Chen et al., 2019).

## Research Model and Process

This experiment takes the classroom legal teaching in the Law School in a University in Shanghai as the experimental object. Two classes are recruited: A and B, with 35 students in each class. The law course scores of the previous semester are the pre-test scores. Class A is the experimental group, which adopts the proposed legal ID. Class B serves as the control group, which adopts the traditional method. The experimental time is one semester, and the results of the law course this semester are used as the post-test results. In order to ensure the internal validity of the experiment, Class A and Class B are parallel classes divided by the school. Students’ scores and learning abilities in class A are similar to those in class B, and the teacher does not disclose the relevant contents of the experiment to the students. In order to improve the external validity, the final examination results are used for the pre-test and post-test results. Neither the relevant contents of the experiment nor additional tests are disclosed to the students.

During the experiment, the teachers of class A should follow two principles to cooperate with the proposed legal ID:

(1) The principle of feedback control. Teachers should take the teaching activity itself as the object of consciousness, understand and analyze the students’ learning situation promptly, and monitor, reflect, and evaluate the classroom teaching. The goal is to work effectively. According to the actual situation, teachers strengthen, help and guide students promptly and control and adjust the teaching process. Additionally, they should guide students to learn self-feedback and monitoring and cultivate students’ ability and habit of monitoring teaching and their learning activities (Alzobiani, 2020; Audley, 2020).
(2) The main body of students and the leading principle of teachers. In teaching activities, teachers should respect the dominant position of students and enable students to participate actively. Additionally, they do not give up the guiding responsibility of teachers and perfectly combine the main body of students with the leading role of teachers (Abuhmaid, 2020).

Class A teachers teach according to the newly designed ID. It is mainly divided into three stages:

In the first stage, students’ legal awareness and perceptual cognition of law will be cultivated based on theoretical legal knowledge, using the case teaching method in combination with audit and internship. At this stage, teachers should guide students to encode abstract conceptual information effectively and help students transfer knowledge;
In the second stage, according to the teaching content, the interactive teaching of the mock court will be carried out in the classroom. As such, on-site interaction allows students to deepen their understanding and memory of abstract legal concepts. Doing so improves students’ basic legal skills and thinking mode and enhances their understanding of their shortcomings through interaction and comparison between groups;
In the third stage, through the internship, legal aid, and other activities, students’ thinking and expression abilities are trained, and students’ sense of social responsibility and dedication are cultivated.

**FIGURE 3:**
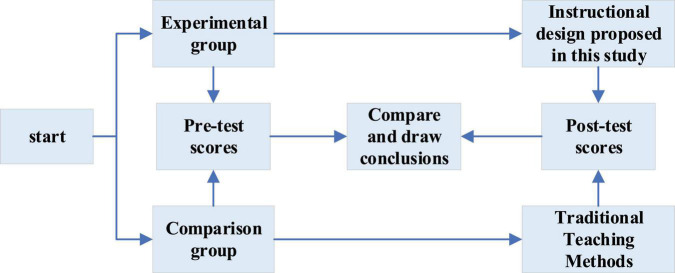
Experimental process.

The specific process of this experiment is unfolded in Figure 3.

## Experimental Results and Analysis

### Experimental Results

**FIGURE 4:**
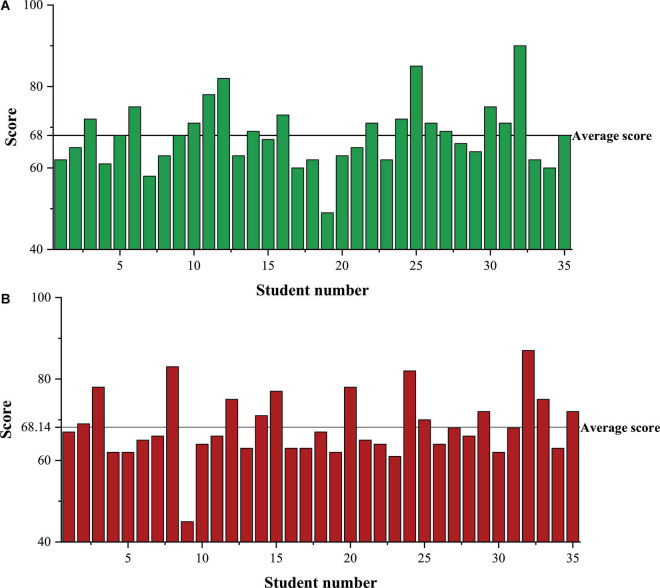
Pre-test scores of students in Class A and B. **(A)** Is the pre-test score of Class A students, **(B)** is the pre-test score of Class B students.

Class A is the experimental group, and Class B is the control group. Class A and Class B each have 35 students, and their law scores in the previous semester are the pre-test scores. The pre-test scores of Classes A and B are shown in Figure 4.

**FIGURE 5:**
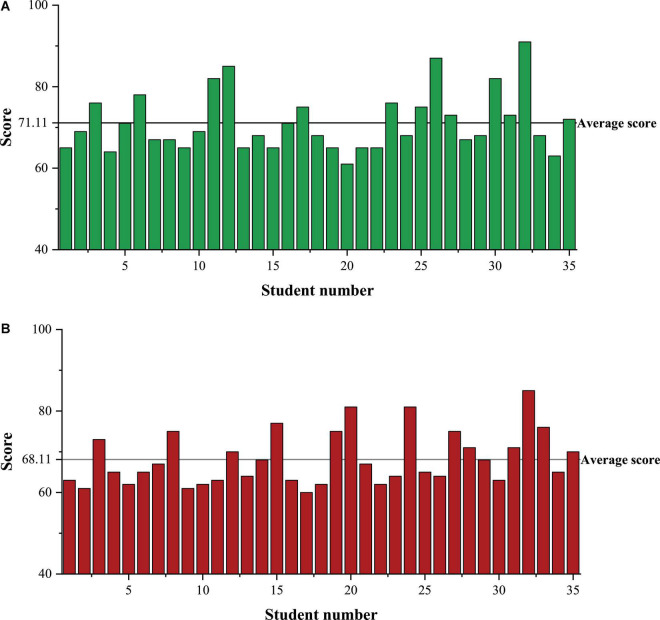
Post-test scores of students in Classes A and B. **(A)** Is the post-test score of Class A, **(B)** is the post-test score of Class B.

In Figure 4, after statistical calculation, the average pre-test score of Class A and Class B is 68 and 68.14. There is very little difference in the pre-test scores between the two classes. The historical scores of Class A and Class B after one semester are post-test scores, as depicted in Figure 5.

In Figure 5, through statistical calculation, the average post-test score of Class A and Class B post-test is 71.11 and 68.11.

### Experimental Analysis

**FIGURE 6:**
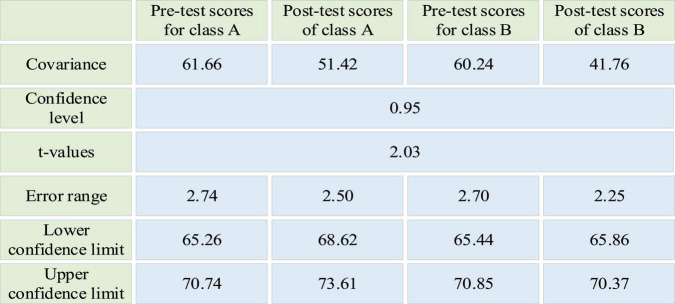
Comparison of pre-and post-test scores of Classes A and B.

Students’ pre-and post-test scores in Classes A and Class B are compared in Figure 6.

In Figure 6, compared with the pre-test score, the covariance of the post-test score of class A has decreased from 61.66 to 51.42. The average score of class A has increased from 68 to 71.11. Thus, the overall score of class A has improved, and the volatility has become smaller. Compared with the pre-test score, the post-test score of class B has decreased from 60.24 to 41.76. The average score has decreased from 68.14 to 68.11. Although the volatility has become smaller, the overall score has decreased. When the confidence level is set to 0.95, the pre-test confidence interval of class A is 65.26–70.74, and the post-test confidence interval is 68.62–73.61. Meanwhile, 95% of the students in class A scored between 65.26 and 70.74 before the experiment. After the experiment, 95% of the students in class A scored between 68.62 and 73.61, a noticeable improvement. Similarly, before the experiment, 95% of the students in class B scored 65.44–70.85. After the experiment, 95% of the students in class B scored 65.86–70.37, without significant improvement. It can be proved that the proposed DL-based legal ID is scientific and effective.

### Discussion

From the perspective of EPSY, this work optimizes the current legal ID through the DL theory. In the traditional legal ID, teachers often focus on theoretical knowledge in books and unilaterally emphasize the logic of law while ignoring its practical experience. At the same time, both teachers and students deem memorization of theories and concepts the most critical task, leading to students’ lack of creative thinking ability. The ID proposed here combines theory with practice. Various learning and practice channels and mock courts in and out of the classroom increase students’ practical ability and deepen their memory of theories. These methods also boost student interest in law and the enterprise spirit that the legal industry should have.

## Conclusion

From the perspective of EPSY, this work optimizes the legal ID in CAUs through DL theory. It takes a Law School in a University in Shanghai as the research base. Firstly, it makes an in-depth understanding of the current situation of college legal teaching in the school through an audit, communication, and investigation. It then integrates the DL theory to find the problems and reasons in the current college legal teaching. Consequently, the ID is carried out from five perspectives: language information, wisdom skills, cognition, action skills, and attitude. Finally, the ID is applied to practice. Further, 70 students in Class A and Class B of the University are selected as the experimental and control groups to test the proposed legal ID. The pre-test and post-test scores are compared. Class A and Class B are parallel classes assigned by the school with similar academic scores. The teacher does not disclose the relevant contents of the experiment to the students. The pre-test and post-test scores of the experiment take from the final examination. After one semester, the average legal score of class A has increased from 68 to 71.11. The covariance has decreased from 61.66 to 51.42. Before the experiment, 95% of the students in class A scored between 65.26 and 70.74. After the experiment, 95% of students in class A scored between 68.62 and 73.61. Thus, the overall score of class A has increased. The average legal score of class B has decreased from 68.14 to 68.11, and the covariance has decreased from 60.24 to 41.76. Before the experiment, 95% of the students in class B scored 65.44–70.85. By comparison, after the experiment, 95% of the students in class B scored 65.86–70.37, without significant improvement. Although the covariance of class B is lower than class A, its average score has decreased, indicating a decreased overall score. Therefore, the proposed DL-based legal ID is scientific and effective. Lastly, the proposed legal ID is not perfect. The future work will optimize the proposed design to enable students to develop more comprehensively. In general, this work has certain reference significance for optimizing the ID of CAU and improving the comprehensive quality of college-student talents.

## Data Availability Statement

The raw data supporting the conclusions of this article will be made available by the authors, without undue reservation.

## Ethics Statement

The studies involving human participants were reviewed and approved by Shanghai International Studies University Ethics Committee. The patients/participants provided their written informed consent to participate in this study. Written informed consent was obtained from the individual(s) for the publication of any potentially identifiable images or data included in this article.

## Author Contributions

Both authors listed have made a substantial, direct, and intellectual contribution to the work, and approved it for publication.

## Conflict of Interest

The authors declare that the research was conducted in the absence of any commercial or financial relationships that could be construed as a potential conflict of interest.

## Publisher’s Note

All claims expressed in this article are solely those of the authors and do not necessarily represent those of their affiliated organizations, or those of the publisher, the editors and the reviewers. Any product that may be evaluated in this article, or claim that may be made by its manufacturer, is not guaranteed or endorsed by the publisher.
