# Supplementary material for: Legal Instructional Design by Deep Learning Theory Under the Background of Educational Psychology
Source: Front Psychol. 2022 Jul 19;13:917174. doi: 10.3389/fpsyg.2022.917174 (PMC9343705; doi:10.3389/fpsyg.2022.917174)
Supplement: Supplementary file 1 [file Data_Sheet_1.docx]

**Appendix 1**

Pre-test questions

I. Fill in the blanks (1 point for each blank, 20 points in total)

1. Criminal law is the law that stipulates ( ), ( ), and ( ).

2. According to the requirements of the principle of adaptation of crime, responsibility, and punishment, the severity of punishment should be commensurate with the criminal's ( ) and ( ).

3. The principle of China's criminal law to solve the problem of retroactivity is ( ).

4. The constitution of a crime is an organic unity of all the subjective and objective elements required by the criminal law to determine the specific act ( ) and ( ).

5. If a unit commits a crime, it shall be sentenced to ( ) punishment. Those who are directly responsible for the crime shall be sentenced to ( ) and ( ) and ( ) punishment.

6. ( ) or ( ) to commit a crime are the crime preparation.

7. Implicated crime refers to the criminal pattern in which the actor commits a certain crime (this crime), ( ), or commits other crimes (other crimes).

8. The subject of penalty application is ( ).

9. The death penalty shall not be applied to those who ( ) when committed crimes or to those who ( ) when being tried.

10. The object of parole is the criminals who have been sentenced to ( ) and ( ).

II. Multiple choice questions (each sub-question has at least one correct answer. Please fill in the serial number letters of the correct answers in the brackets of the questions. No score will be given for s wrong, multiple, and less choice. 2 points for each topic, 20 points in total)

1. Compared with other department laws, criminal law has the following remarkable characteristics ( ).

A. The most theoretical and practical B. The most stringent mandatory

C. The scope of social relations protected is broader D. More means to protect social relations

2. The "proviso" in the provisions of the criminal law means ()

A. Supplement to the preceding paragraph B. Restrictions on the preceding paragraph

C. Emphasis on the preceding paragraph D. Exceptions to the preceding paragraph

3. The implementation time of the revised criminal code of China is ().

A. March 14, 1997 B. July 1, 1997

C. October 1, 1997 D. January 1, 1998

4. There are 12 kinds of specific crimes in the chapter "crime of infringing upon property" in the special provisions of the criminal code of China. The object of common infringement of these crimes belongs to ().

A. General object B. Kindred object of a crime C. Direct object D. Complicated object of the crime

5. The omission constituting a crime is based on the specific obligation of the perpetrator to perform a certain positive act. This specific obligation arises from ()

A. Requirements of social morality B. Job or business requirements

C. Express provisions of law D. Dangerous state caused by the prior behavior of the actor

6. While hunting, A found a child playing next to his prey. He knew he was not good at shooting, but he was eager to hunt, so he fired the gun. As a result, he missed the prey and hit and killed the child. The psychological attitude of behavior belongs to ().

A. Direct intent B. Negligent fault C. Indirect intent D. Overconfidence fault

7. The types of accomplished crimes are ().

A. Behavioral crime B. Potential damage offense C. Consequential offense D. Act crime

8. The act of robbery and murder of A committed two criminal law provisions and two charges. In fact, it only constitutes one crime of robbery. This situation is called () in criminal law theory.

A. Imaginative joinder of offenses B. Overlap of enactments C. Joint offense D. Absorptive offense

9. The executive organ of control is ().

A. People's court B. People's Procuratorate C. Organization of reform-through-labor D. Public security unit

10. Wu was sentenced to life imprisonment and fixed-term imprisonment for three years and fined 10,000 RMB for intentional injury and embezzlement. In deciding on the punishment to be executed, it shall adopt ().

A. Absorption principle B. The principle of restriction and aggravation and the principle of merging

C. Absorption principle and restriction and aggravation principle D. Absorbing principle and merging principle

III. Explanation of terms (4 points for each topic, 20 points in total)

1. Object of a crime 2. Discontinuation of crime 3. Deprivation of political rights 4. Combined punishment for several crimes 5. Execution of punishment

IV. Short answer questions (8 points for each sub-question, 24 points in total)

1. Briefly describe the necessary conditions for the establishment of justifiable defense.

2. Briefly introduce the concept and establishment conditions of joint crime.

3. Briefly introduces the concept of general voluntary surrender and its establishment conditions.

V. Case analysis (16 points) this paper briefly introduces the concept of general voluntary surrender and its establishment conditions.

Facts of the case: Xu, a male, born on October 21, 1984, was a deaf-mute student.

Xu is the only child in his family. Affected by the bad social atmosphere, he has no interest in learning, often plays truant, and even has established a relationship with some "gangsters" outside the school. On the evening of September 17, 2000, Xu told his mother, Zhao, that he did not want to go to school the next day. Seeing that his son wanted to play truant again, Zhao was furious and raised his hand and hit his son twice in the mouth. That night, while Zhao was sleeping, Xu put a bag of rat poison (tetramine) into the traditional Chinese medicine that Zhao must drink daily. After finding 160 RMB in Zhao's wallet, Xu went to the Internet bar to play games. The next morning, Zhao died of poisoning after drinking Chinese medicine mixed with rat poison. When Xu came home to learn that his mother had died, he admitted to his father that he had done it. Under his father's leadership, he surrendered to the police station and explained the whole story.

Try to analyze and explain how Xu's behavior should be recognized and punished?

**Appendix 2**

Post-test questions

I. Fill in the blanks (1 point for each blank, 20 points in total)

1. According to the method of interpretation, criminal law interpretation can be divided into ( ) and ( ).

2. The principle of Chinese criminal law to solve the spacing effect is based on the ( ) principle. It adopts ( ), ( ), and ( ) principles’ reasonable factors.

3. The crime stipulated in the criminal law of China refers to the behavior that ( ) Chinese society violates the criminal law and should be punished by ( ).

4. China's criminal law stipulates that the relative age of criminal responsibility is ( ) years old to ( ) years old.

5. A mentally ill person who causes harmful results in ( ) or in ( ) his own conduct and who has been identified and confirmed through legal procedures shall not bear criminal responsibility.

6. Excessive defense refers to ( ) in the process of justifiable defense, which causes obviously ( ) and should be held criminally responsible according to law.

7. The purpose of the penalty is to prevent crime, including ( ) and ( ).

8. The term of criminal detention shall be not more than ( ) and no less than ( ).

9. For ( ), probation and parole are not applicable.

10. The term of life imprisonment commuted to fixed-term imprisonment shall be counted from the date of ( ).

II. Multiple choice questions (each sub-question has at least one correct answer. Please fill in the serial number letters of the correct answers in the brackets of the questions. No score will be given for s wrong, multiple, and less choice. 2 points for each topic, 20 points in total)

1. The "proviso" in the provisions of the criminal law means ()

A. Supplement to the preceding paragraph B. Restrictions on the preceding paragraph

C. Emphasis on the preceding paragraph D. Exceptions to the preceding paragraph

2. The principle of China's criminal law to solve the problem of retroactivity is ().

A. Follow the old principle B. Follow the new principle C. Follow the principle of "old and light" D. Follow the principle of innovation and leniency

3. In the theory of criminal law, based on ( ) division standard, the object of crime can be divided into the general object, similar object, and direct object.

A. Number of criminal acts B. Scope of social relations violated by criminal acts

C. Number of times criminal acts infringe on specific social relations D. The number of specific social relations directly violated by criminal acts

4. A person who commits a crime after physical intoxication belongs to ().

A. Person with full criminal responsibility B. Person without criminal responsibility

C. Relatively incapable of criminal responsibility D. Person with ability to mitigate criminal responsibility

5. In the course of committing a crime, party A voluntarily gave up the crime without causing damage. A should be given ( ).

A. Exemption from punishment B. Mitigation or exemption from punishment C. Exemption or mitigation of punishment D. Mitigation of punishment

6. The ringleaders who organize and lead criminal groups shall be punished in accordance with () for all crimes.

A. The group committed the crime B. He organized and led the crime C. He was involved in the crime D. He commanded the crime

7. In order to force Party B to repay his debt, Party A illegally detained Party B for ten days. The behavior of A belongs to ().

A. Continuous offense B. Implicated offender C. Continuing offense D. Consequential aggravated offense

8. In imposing a fine on a criminal, the amount of the fine shall be determined according to his/her ().

A. Nature of crime B. Criminal circumstances C. Proceeds of crime D. Family economic status

9. The minimum probation period for suspension of fixed-term imprisonment shall be ().

A. Two months B. Six months C. One year D. Two year

10. The time limit for prosecution of a crime with a statutory maximum punishment of not less than 10 years' imprisonment is ().

A. Ten years B. Fifteen years C. Twenty years D. Twenty-five years

III. Explanation of terms (4 points for each topic, 20 points in total)

1. Age of criminal responsibility 2. Criminal Negligence 3. Joint crime 4. Surrender oneself 5. Lapse of time

IV. Short answer questions (8 points for each sub-question, 24 points in total)

1. Briefly introduce the concept and characteristics of the crime constitution.

2. Briefly describe the restrictive provisions of the general criminal law provisions on the death penalty application.

3. Briefly describe the conditions applicable to parole.

V. Case analysis (16 points)

Facts of the case: Wang, male, 23, a factory worker

Wang found a temporary job in the factory set up by Jiang in 2004. In the middle of April 2005, Jiang dismissed Wang on the ground that Wang stole things from his unit and refused to pay him his salary from February to April. Wang refused to accept it. He negotiated with Jiang several times, but both sides broke up unhappily because they insisted on their own words. On April 23, Mr. Wang talked with Mr. Jiang again. As a result, there was a dispute. The two sides fought. Mr. Jiang, who was tall and powerful, also punched Mr. Wang. Wang, who felt that he had suffered a loss, took revenge. At noon on April 24, Wang appeared in front of Jiang's house with a barrel of gasoline. Jiang opened the door and found that it was Wang. He asked Wang what was wrong with him. Wang said, "it is still about salary. You have to give me a satisfactory answer today, or you will not be able to eat your fruit!" Wang shook the gasoline barrel in his hand as he said. As soon as Jiang smelled the smell of gasoline, he immediately returned to the house, locked the door, and called the security guard. Wang shouted abuse outside the door and poured gasoline on Jiang's door. When the security guard arrived, he just heard Wang say, "if you do not come out again, I will burn you!" He immediately threw Wang to the ground and found a lighter in his pocket.

Please analyze and explain the reason: what is the nature of Wang's behavior? What should be done?
